# Interactions between oxytocin receptor gene and intergroup relationship on empathic neural responses to others’ pain

**DOI:** 10.1093/scan/nsz029

**Published:** 2019-04-24

**Authors:** Siyang Luo, Ting Zhang, Wenxin Li, Meihua Yu, Grit Hein, Shihui Han

**Affiliations:** 1 School of Psychological and Cognitive Sciences, PKU-IDG/McGovern Institute for Brain Research, Beijing Key Laboratory of Behavior and Mental Health, Peking University, Beijing, China; 2 Department of Psychology, Guangdong Key Laboratory of Social Cognitive Neuroscience and Mental Health, and Guangdong Provincial Key, Laboratory of Brain Function and Disease, Sun Yat-Sen University, Guangzhou, China; 3 Department of Psychiatry, Psychosomatic and Psychotherapy, University of Würzburg, Würzburg, Germany

**Keywords:** empathy, pain, racial bias, oxytocin receptor gene, ERP

## Abstract

Empathic neural responses to others’ suffering are subject to both social and biological influences. The present study tested the hypothesis that empathic neural responses to others’ pain are more flexible in an intergroup context in G/G than A/A carriers of the oxytocin receptor gene (OXTR) (rs53576). We recorded event-related brain potentials to painful *vs* neutral expressions of Asian and Caucasian faces that were assigned to a fellow team or an opponent team in Chinese carriers of G/G or A/A allele of OXTR. We found that G/G carriers showed greater neural responses at 136–176 ms (P2) over the frontal/central region to painful *vs* neutral expressions of faces with shared either racial or mini group identity. In contrast, A/A carriers showed significant empathic neural responses in the P2 time window only to the faces with both shared racial and mini group identity. Moreover, the racial in-group bias in empathic neural responses varied across individuals’ empathy traits and ethnic identity for G/G but not A/A carriers. Our findings provide electrophysiological evidence for greater flexibility of empathic neural responses in intergroup contexts in G/G (*vs* A/A) carriers of OXTR and suggest interactions between OXTR and intergroup relationships on empathy for others’ suffering.

## Introduction

Group living is a human universal that is characterized by a preference of one’s in-group members in both behavior and psychological needs (Brown, 1991; Kurzban and Neuberg, 2005). Recent brain imaging research has documented substantial influences of intergroup relationships on brain activity related to social cognition and social behavior (Cikara and Van Bavel, 2014). Specifically related to the current work is the finding of an in-group bias in brain activities in response to others’ suffering or empathic neural responses (Eres and Molenberghs, 2013; Han, 2018) that play a key role in pro-social behavior (Decety and Jackson, 2004; Decety *et al.*, 2016). Because individuals live in multiple social groups (Tajfel, 1982), researchers have examined extensively whether and how intergroup relationships defined in one way or another modulate empathic neural responses to others’ pain.

One line of research tested how interracial relationships modulate empathic neural responses. An early functional magnetic resonance imaging (fMRI) study found that, while watching painful *vs* non-painful stimulations applied to others activated the anterior cingulate cortex (ACC) and supplementary motor area (SMA), the ACC/SMA activation was decreased in response to painful stimulations applied to other-race than same-race models in both Chinese and white students (Xu *et al.*, 2009). fMRI studies also revealed decreased anterior insular activity in responses to painful (*vs* neutral) expressions of other-race than same-race faces (Sheng *et al.*, 2014). The racial in-group bias in empathy (RIBE) is also evident in electrophysiological responses. Event-related brain potential (ERP) studies have repeatedly shown that, while painful (*vs* neutral) expressions increased the amplitude of a positive component peaking ~160 ms after stimulus onset over the frontal/central regions (P2), the P2 amplitude to painful (*vs* neutral) expressions, which predicted subjective feelings of others’ pain (Sheng and Han, 2012), was significantly larger when perceiving same-race than other-race faces (Sheng and Han, 2012; Sheng *et al.*, 2013, 2016; Li *et al.*, 2015; Han *et al.*, 2016). RIBE indexed by brain activity has been replicated using fMRI, ERP and motor evoked potential measures by different research groups that tested Asian, white and black participants in Asia (Xu *et al.*, 2009; Sheng and Han, 2012; Sheng *et al.*, 2013, 2016; Li *et al.*, 2015; Han *et al.*, 2016), Europe (Avenanti *et al.*, 2010; Azevedo *et al.*, 2013; Sessa *et al.*, 2014), North America (Mathur *et al.*, 2010; Cheon *et al.*, 2011), Australia (Contreras-Huerta *et al.*, 2013) and South Africa (Fourie *et al.*, 2017). These findings indicate pervasive effects of interracial relationships on neural activities underlying empathy for pain (see Han, 2015, 2018, for review).

Another line of research on the in-group bias in empathy has employed minimal group manipulation to investigate the effect of intergroup relationships on empathic neural responses (see Cikara *et al.*, 2011a and Molenberghs, 2013, for review). For example, an fMRI study discovered that the left anterior insula showed stronger activation when soccer fans witnessed a fan of their favorite team (*vs* a rival team) experiencing pain (Hein *et al.*, 2010). In addition, the in-group *vs* out-group difference in anterior insular responses predicted the extent of participants’ in-group favoritism in subsequent costly helping. Another fMRI study found that witnessing failure of the favored team or success of the rival team was associated with activations in the ACC and insula whereas watching success of the favored team or failure of the rival team was associated with activations in the key node of the reward system (e.g. the ventral striatum) (Cikara *et al.*, 2011b).

These brain imaging findings provide evidence that empathic neural responses related to pro-social behavior are sensitive to intergroup relationships based on racial identity and mini group (e.g. soccer team) identity and are stronger toward in-group than out-group members’ pain. The results raise a critical issue of whether mini group relationships can overcome interracial relationships in modulating empathic neural responses. To clarify this question is important for understanding the in-group bias in empathy and social behavior from two perspectives. Theoretically, to elucidate whether and how different social group memberships interact to modulate empathic neural responses is critical for understanding the dynamic affective and neural processes that underlie adaptive social behavior in societies consisting of multiple social groups (Tajfel, 1982). Practically, if mini group relationships that are built temporarily can override interracial relationships in modulating empathic neural responses, RIBE may be reduced by mini group manipulation as a potential intervention for the racial bias in pro-social behavior.

Recent neuroimaging studies have examined the interaction of racial identity and mini group identity on empathic neural responses but reported inconsistent results. An early ERP study employed the minimal group paradigm to examine empathic neural responses in a mixed group relationship context where participants were assigned to a team consisting of both same-race and other-race individuals to compete with another team that also consisted of same-race and other-race individuals (Sheng and Han, 2012). Under this condition, each individual had a group identity identified by interracial or inter-mini group relationships. It was found that the P2 amplitude to painful (*vs* neutral) expressions was greater to same-race than other-race faces from the opponent team but were comparable to same-race and other-race faces from the fellow team. These findings suggest that the in-group membership defined by mini group relationship may override the interracial relationship to reduce RIBE. A recent fMRI study further reported greater insular activations to perceived pain of in-group *vs* out-group members consisting of both same-race and other-race individuals (Shen *et al.*, 2018). However, another fMRI work that manipulated both interracial and mini group relationships found significant racial in-group bias in ACC and insular activity in response to others’ pain but failed to observe reliable minimal group effects on empathic neural responses (Contreras-Huerta *et al.*, 2014).

Among the multiple (including cognitive, sociocultural and biological) factors that contribute to RIBE, oxytocin has been suggested to be a critical biological underpinning (Sheng *et al.*, 2013; Han, 2018). An ERP study found that intranasal administration of oxytocin significantly increased P2 amplitudes to painful (*vs* neutral) expressions of same-race faces but did not increase that of other-race faces (Sheng *et al.*, 2013). These ERP results are consistent with behavioral findings that oxytocin induced greater trust and more cooperation toward in-group rather than reduced those toward out-group in competitive situations (De Dreu *et al.*, 2010; Van IJzendoorn and Bakermans-Kranenburg, 2012). Behavioral studies also discovered that individuals who carry G alleles of a single nucleotide polymorphism (rs53576) in the oxytocin receptor gene (OXTR) tended to exhibit greater empathic parenting, empathic accuracy and empathic concern relative to those who carry A alleles of OXTR (rs53576) (Bakermans-Kranenburg and van IJzendoorn, 2008; Rodrigues *et al.*, 2009; Tost *et al.*, 2010; Kogan *et al.*, 2011; Krueger *et al.*, 2012; Smith *et al.*, 2014). Recent fMRI findings further revealed associations between RIBE and OXTR (rs53576) such that carriers of G compared to A alleles of OXTR rs53576 showed a greater racial in-group bias in empathic response in the ACC (Luo *et al.*, 2015a). In addition, both behavioral and fMRI findings suggest a stronger association between cultural orientations (e.g. interdependence) and empathy trait/empathic neural responses in G allele carriers compared to A/A homozygotes of OXTR rs53576 (Luo *et al.*, 2015b).

While these results suggest that G compared to A allele carriers of OXTR rs53576 are more sensitive to social relationships and cultural experiences in their empathic responses to others’ pain, two hypotheses arise from the previous findings regarding the potential interactions between OXTR and intergroup relationships on empathy. First, if empathy in G (*vs* A) allele carriers is more flexible in response to social relationships, in the context of mixed intergroup relationships, G carriers may show empathic responses to perceived pain of in-group members defined by any intergroup relationships. Applying this hypothesis to the interactions of interracial and mini group relationships on empathy, we predicted that G carriers should show increased neural responses to perceived painful (*vs* neutral) expressions of in-group members defined by either one or both of the two intergroup relationships (i.e. individuals of same-race or individuals from the fellow team). In other words, G carriers show no empathic neural responses only to those who are perceived as out-group members in terms of both interracial and mini group relationships. Second, if empathy in A (*vs* G) allele carriers is less flexible in response to social relationships, in the context of mixed intergroup relationships, these individuals may show empathic neural responses to perceived painful (*vs* neutral) expressions of those who are in-group members in terms of both interracial and mini group relationships.

To test these hypotheses, we recorded ERPs from Chinese adults homozygous for A (A/A) or G (G/G) allele of OXTR rs53576 while they viewed painful or neutral expressions of Asian and Caucasian faces. Using the minimal group paradigm half of these faces were assigned to the fellow team and half to the opponent team. Critically, both the fellow and opponent teams consisted of half Asian and half Caucasian faces. This design allowed us to investigate how interracial and inter-mini group relationships interact to modulate empathic neural responses in a specific time window by examining ERPs to painful (*vs* neutral) expressions of same-race and other-race faces from the fellow and opponent teams. We focused on the P2 amplitude that has been shown to be enlarged by painful (*vs* neutral) expressions and can predict subjective feelings of others’ pain (e.g. Sheng and Han, 2012). As expected, we found evidence for specific patterns of modulation of the P2 amplitude in response to others’ pain by interactions of OXTR and intergroup relationships.

## Methods

### Participants

The current study recruited 50 Chinese female university students including 25 G/G carriers and 25 A/A carriers from a genotyped sample of 1532 subjects from our previous study (Luo *et al.*, 2015b). The sample size for the current work was determined based on our previous research that showed robust evidence for a racial in-group bias in ERP amplitudes in response to painful expressions (Sheng and Han, 2012) and was selected to allow us to detect a medium effect size for a within–between interaction with 90% power (*f* = 0.25, with alpha = 0.05 and power = 0.90, calculated using the G*Power software, Faul *et al.*, 2007). All participants were right handed, had normal or corrected-to-normal vision and reported no abnormal neurological history. Age and trait empathy scores were matched between the two genotyped groups (*P*s > 0.05, Table 1). Informed consent was obtained from all participants before electroencephalograph (EEG) recording. This study was approved by a local ethics committee at the School of Psychological and Cognitive Sciences of Peking University.

**Table 1:** Age, empathy traits and ethnic identity of G/G and A/A carriers in this study

	G/G homozygote		A/A homozygote	
Age	21.2	± 2.24	21.8	± 2.73
IRI	69.8	± 10.42	70.2	± 10.50
Perspective taking	18.7	± 3.67	19.2	± 3.39
Empathic concern	17.4	± 3.20	18	± 3.56
Fantasy	18.3	± 4.91	18.2	± 3.97
Personal distress	15.4	± 3.32	14.8	± 3.39
Ethnic identity	32.6	± 7.19	33.6	± 5.52

IRI, Interpersonal Reactivity Index Scale.

### Stimuli and procedure

Stimuli were adopted from our previous work (Sheng and Han, 2012), consisting of digital photographs of faces with neutral or pain expressions of 16 Asian models (8 males) and 16 Caucasian models (8 males). Each model contributed two face images, one with a neural expression and one with a painful expression. Pain intensity, luminance levels and attractiveness were matched between Asian and Caucasian faces (Sheng and Han, 2012).

Before EEG recording, the participants were informed that they had been randomly assigned to the blue or green team for a competitive game and that they had to remember all of the faces of fellow team and opponent team members. Eight Asian and eight Caucasian faces (half males and half females) were assigned to the fellow team, and other faces were assigned to the opponent team. The participants completed three learning tasks to remember the fellow team and opponent team members marked by their T-shirt colors (Sheng and Han, 2012) before EEG recordings. The intensity of painful expressions was matched between fellow and opponent team faces. To ensure that the participants believed in the existence of the game, a female confederate was present and assigned to the opponent team. Both the participant and the confederate were asked to wear a blue or green T-shirt. In the first learning task, the participants were presented with neutral faces of all the models in colored T-shirts simultaneously and were asked to learn and remember the fellow team and opponent team members. To avoid the possibility that participants adopted a strategy of remembering only fellow team members, the participants were told that a third team would appear in a subsequent procedure, so it was very important that they had to remember the members of both teams. The first learning task lasted for ~5 min.

In the second learning task, faces of fellow team and opponent team members were presented on the left and right (or the reverse) sides of the screen, respectively. The participants were asked to search for a target face in a face array consisting of the fellow team and opponent team members by moving a frame around one of the faces. Each participant completed 4 blocks of 32 trials. In Blocks 1 and 2, the target face and the faces in the search array were matched in expression (neutral or painful). In Blocks 3 and 4, the target face and faces in the search array were different in expression (e.g. searching for a target with a painful expression in an array of faces with neutral expressions or the reverse).

In the third learning task, each face without a colorful T-shirt was presented on a screen until the participant pressed a button to categorize the face as a fellow team or opponent team member. Each participant completed two blocks of categorization tasks. Each face appeared once in each block, and feedback was given after each trial. Each participant was given a memory test before and after the EEG session. The procedure of the memory test was identical to the third learning task except that the participants performed only one block of trials without feedback.

During EEG recordings, the participants were presented with faces of Asian and Caucasian models in blue or green T-shirts (representing fellow or opponent teams) and performed race judgments (Asian *vs* Caucasian) in 8 blocks of 128 trials. Each block started with an instruction to define the task. Each face was displayed for 200 ms in the center of a gray background with a visual angle of 3.8 degrees × 4.7 degrees (width × height: 7.94 × 9.92 cm) at a viewing distance of 120 cm. The inter-stimulus intervals consisted of a fixation cross with a duration that randomly varied between 800 and 1400 ms. The participants responded to each stimulus by pressing the left or right button using the left or right index finger.

After EEG recording, the participants rated the intensity of pain portrayed by each face and their subjective feelings of unpleasantness induced by each face on a 9-point Likert scale (1 = not at all, 9 = extremely unpleasant). The participants were also asked to complete a race version of the implicit association test (IAT, Greenwald *et al.*, 1998) to estimate their implicit attitudes toward Asian and Caucasian faces. They categorized Asian faces/positive words with one key and Caucasian faces/negative words with another key in two blocks and Asian faces/negative words with one key and Caucasian faces/positive words with another key in another two blocks. A D score, calculated based on an established algorithm for response latencies (Greenwald *et al.*, 2003), provided an index of participants’ implicit attitudes toward racial in-group and out-group faces. A D score larger than zero indicates that in-group faces are associated with a positive rather than a negative attitude compared with out-group faces. The participant also completed the Interpersonal Reactivity Index (IRI, Davis, 1983) and the Multigroup Ethnic Identity Measure (Phinney, 1992) to measure their empathy traits and self-recognized ethnic identity.

### EEG recording and analysis

The EEG was continuously recorded using the NeuroScan system from 62 scalp electrodes that were mounted on an elastic cap in accordance with the extended 10–20 system and were referenced to the average of the left and right mastoid electrodes. The electrode impedance was maintained <5 kΩ. Eye blinks and vertical eye movements were monitored with electrodes located above and below the left eye. The horizontal electro-oculogram was recorded from electrodes placed 1.5 cm lateral to the left and right external canthi. The EEG was amplified (bandpass 0.1–100 Hz) and digitized at a sampling rate of 250 Hz. EEG data analysis was conducted using Scan 4.5 software. The ERPs in each condition were averaged separately off-line with an epoch beginning 200 ms before stimulus onset and continuing for 1200 ms. Trials contaminated by eye blinks, eye movements, muscle potentials exceeding ±50 μV at any electrode or response errors were excluded from the average. This resulted in rejection of 21.3% ± 9.9% of the trials. The baseline for the measurements of ERP amplitudes was the mean voltage of a 200 ms pre-stimulus interval, and the time windows for the measures referred to stimulus onset. To avoid potential significant but bogus effects on ERP amplitudes due to multiple comparisons (Luck and Gaspelin, 2017), the mean amplitudes of ERP components were calculated at electrodes according to the peak distribution of each component in voltage topographies. These included Fz, FCz, F3–F4 and FC3–FC4 for the P2 and N2 components; FCz, Cz, CPz, FC1–FC2, C1–C2 and CP1–CP2 for the P3 component. When selecting the time window for measuring the mean amplitude of an ERP component, we checked the peak latency of the grand average across G/G and A/A groups and the peak latencies for G/G and A/A groups separately. If the peak latency of the grand average across the two groups was the same as that of the G/G and A/A groups (e.g. for the P2 and N2 components), we used the same time window to calculate the mean amplitude. Otherwise, we calculated the mean amplitude around the peak latency of an ERP component separately for each group (e.g. for the P3 component). Reaction times (RTs), response accuracies and mean ERP amplitudes were subject to repeated measure analyses of variance (ANOVAs) with mini group (fellow *vs* opponent team), expression (painful *vs* neutral) and race [Same-race (Asian) *vs* Other-race (Caucasian)] as within-subjects variables and genotype (G/G *vs* A/A) as a between-subjects variable.

Both voltage topography and the standardized low resolution brain electromagnetic tomography (sLORETA) (Pascual-Marqui, 2002) were used to estimate potential sources of empathic neural responses. sLORETA is a linear method of computing statistical maps from EEG data that estimates the locations of the underlying source processes and does not require a priori hypotheses regarding the field distribution of the active sources. We performed the analysis using sLORETA to assess the potential 3D current sources of neural activity that differentiated between ERPs to painful and neutral expressions. A boundary element model was first created with approximately 5000 nodes from a realistic head model. Statistical non-parametric mapping was calculated in a specific time window to estimate the source that differentiated ERPs to painful and neutral expressions. The log of the *F* ratio of averages was used and considered with a 0.95 level of significance.

## Results

### Behavioral results

The results of the memory tests of fellow and opponent team members before and after EEG recordings were significantly higher than the chance level (50%) (before EEG recording: G/G: 81.2 ± 12.9%; A/A: 82.7 ± 12.4%; after EEG recording: G/G: 78.6 ± 12.4%; A/A: 80.7 ± 12.4%; *P*s < 0.001). IAT D scores did not differ significantly from zero for either G/G or A/A genotype group [0.04 ± 0.23 and 0.03 ± 0.17; *t*(25) = 0.98 and 1.00; *P*s > 0.3], suggesting that neither G/G or A/A carriers had implicit negative attitude toward other-race faces. Self-reported scores of ethnic identity did not differ significantly between the two genotype groups [*t*(48) = 0.55, *P* = 0.584].

The ANOVA of RTs during EEG recording showed a significant main effect of expression [*F*(1,48) = 13.47, *P* = 0.001, η_p_^2^ = 0.22, Table 2] due to slower responses to painful than neutral expressions. There was a significant interaction of expression–race [*F*(1,48) = 11.36, *P* = 0.001, η_p_^2^ = 0.19] because, relative to neutral expressions, painful expression of Asian faces [*F*(1,48) = 29.72, *P* < 0.001, η_p_^2^ = 0.38] but not Caucasian faces [*F*(1,48) = 0.07, *P* = 0.79, η_p_^2^ = 0.001] slowed responses during race judgments, replicating the previous finding (Sheng and Han, 2012). The ANOVA of response accuracies found a significant main effect of Race [*F*(1,48) = 9.78, *P* < 0.01, η_p_^2^ = 0.17] due to higher accuracy of race judgments for Asian than Caucasian faces. There was also a significant expression–race interaction on response accuracies [*F*(1,48) = 7.91, *P* < 0.01, η_p_^2^ = 0.14]. Further analyses revealed less accurate race judgments on painful (*vs* neutral) expressions for Asian faces [F(1,48) = 6.56, *P* < 0.05, η_p_^2^ = 0.12] but not for Caucasian faces [*F*(1,48) = 2.36, *P* = 0.13, η_p_^2^ = 0.05]. However, there was no significant interaction between mini group (or genotype) and other variables (*P*s > 0.05), indicating comparable task difficulties for G/G and A/A carriers. Participants reported greater pain intensity and self-unpleasantness related to painful *vs* neutral expressions, and rating scores of pain intensity were slightly higher for Caucasian than Asian faces, but these effects did not differ significant between G/G and A/A groups (Table 3).

**Table 2:** RTs and response accuracies of G/G and A/A carriers during EEG recording (mean ± s.d.)

			G/G homozygote		A/A homozygote	
			Painful	Neutral	Painful	Neutral
Accuracy (%)	Fellow team	Asian	91.5 ± 7.7	93.1 ± 7.4	92.9 ± 4.9	93.9 ± 3.9
		Caucasian	90.3 ± 7.9	90.8 ± 7.0	91.0 ± 6.1	90.8 ± 6.1
	Opponent team	Asian	90.4 ± 6.5	91.3 ± 9.3	93.5 ± 3.6	93.7 ± 4.5
		Caucasian	89.9 ± 11.2	89.3 ± 10.8	91.4 ± 5.4	90.2 ± 6.8
RT (ms)	Fellow team	Asian	532.1 ± 59.9	526.1 ± 62.6	557.5 ± 55.1	551.2 ± 49.9
		Caucasian	524.1 ± 65.0	524.6 ± 70.9	553.1 ± 52.8	551.8 ± 54.3
	Opponent team	Asian	536.8 ± 65.5	524.2 ± 57.6	561.6 ± 52.7	554.1 ± 49.7
		Caucasian	520.9 ± 63.0	520.3 ± 57.5	549.8 ± 52.6	553.1 ± 52.4
			**Accuracy**		**RT**	
			***F***	***P***	***F***	***P***
Expression			9.78	0.003	2.69	0.11
Race			2.43	0.13	13.47	0.001
Mini group			1.89	0.18	0.00	0.99
Expression–race			7.91	0.007	11.36	0.001
Expression–mini group			0.005	0.95	1.04	0.31
Race–mini group			3.42	0.07	0.23	0.63
Expression–race–mini group			0.09	0.77	1.28	0.26
Genotype			0.62	0.44	3.21	0.08
Genotype–race			0.63	0.43	0.20	0.66
Genotype–mini group			2.29	0.14	0.47	0.50
Genotype–expression			2.43	0.13	0.70	0.41
Genotype–mini group–expression			0.00	0.99	1.44	0.24
Genotype–race–expression			0.00	0.99	0.06	0.80
Genotype–mini group–race			0.13	0.72	0.006	0.94
Genotype–mini group–race–expression			0.01	0.92	0.001	0.97

**Table 3:** Subjective rating scores in G/G and A/A carriers (mean ± s.d.)

			G/G homozygote		A/A homozygote	
			Painful	Neutral	Painful	Neutral
Pain intensity	Fellow team	Asian	5.76 ± 1.23	1.39 ± 0.80	6.24 ± 1.56	1.49 ± 0.67
		Caucasian	6.18 ± 1.20	1.35 ± 0.88	6.45 ± 1.42	1.45 ± 0.71
	Opponent team	Asian	5.72 ± 1.34	1.38 ± 0.86	6.10 ± 1.51	1.45 ± 0.48
		Caucasian	6.16 ± 1.32	1.36 ± 0.97	6.40 ± 1.37	1.35 ± 0.50
Self-unpleasantness	Fellow team	Asian	4.46 ± 1.72	2.31 ± 1.25	4.76 ± 1.95	2.30 ± 1.14
		Caucasian	4.30 ± 1.83	1.94 ± 0.94	4.79 ± 1.76	2.07 ± 0.99
	Opponent team	Asian	4.56 ± 1.72	2.32 ± 1.32	5.15 ± 1.65	2.45 ± 1.33
		Caucasian	4.32 ± 1.75	2.07 ± 1.27	4.94 ± 1.77	2.43 ± 1.24
			Pain intensity		Self-unpleasantness	
			*F*	*P*	*F*	*P*
Expression			516.51	<0.001	85.31	<0.001
Race			17.57	<0.001	7.83	0.007
Mini group			2.03	0.16	6.93	0.01
Expression–race			22.97	<0.001	0.69	0.41
Expression–mini group			0.002	0.96	0.005	0.95
Race–mini group			0.38	0.54	0.005	0.95
Expression–race–mini group			0.03	0.86	4.39	0.04
Genotype			0.77	0.39	1.08	0.30
Genotype–race			1.19	0.28	1.27	0.27
Genotype–mini group			1.15	0.29	2.51	0.12
Genotype–expression			0.53	0.47	0.44	0.51
Genotype–mini group–expression			0.16	0.69	0.005	0.95
Genotype–race–expression			1.61	0.21	0.21	0.65
Genotype–mini group–race			0.11	0.74	0.02	0.88
Genotype–mini group–race–expression			0.01	0.92	0.66	0.42

### Electrophysiological results

**Fig. 1:**
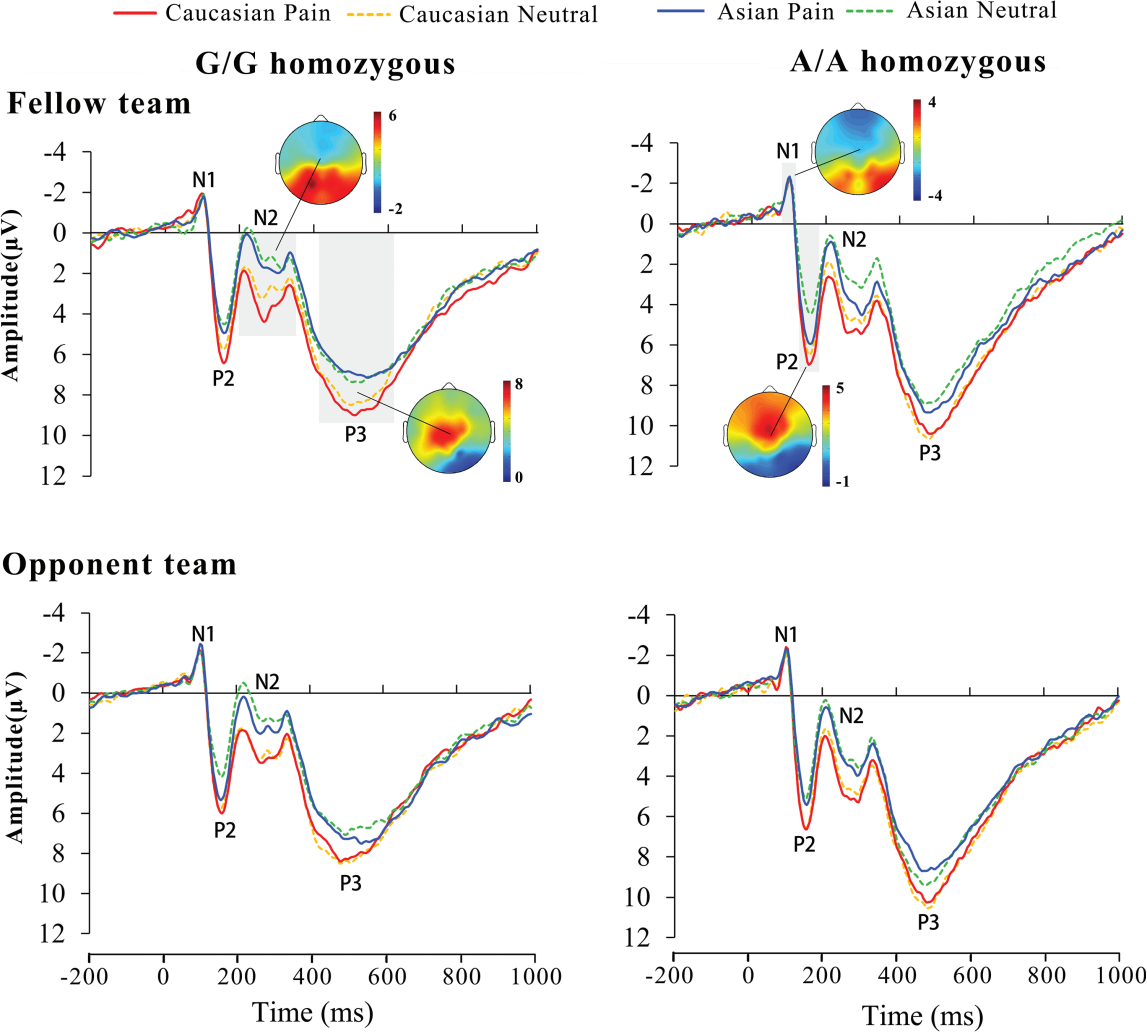
Illustration of the ERPs to painful and neutral expressions of Asian and Caucasian faces at electrode Cz in G/G and A/A carriers, respectively. Gray bars indicate the time windows for measuring the mean amplitude of a specific ERP component. Voltage topographies show the scalp distributions of each ERP component.

Figure 1 illustrates the ERPs to painful and neutral expressions of Asian and Caucasian faces. Similar to the results of the previous work (Sheng and Han, 2012), the ERPs were characterized by a negative wave at 92–120 ms (N1) and a positive deflection at 136–176 ms (P2) over the frontal/central areas, which were followed by a negative wave at 200–340 ms (N2) over the frontal/central region. The peak latencies of these ERP components were similar for G/G and A/A groups. There was a long-latency positivity (P3) with the maximum amplitude over the central/parietal area, which, however, peaked at 460–504 ms for A/A but at 480–564 ms for G/G. Similar numbers of trials with correct responses were included for analyses of ERP amplitudes in G/G and A/A genotype groups (G/G: 100.5 ± 12.5 *vs* A/A: 101.4 ± 13.9, *P* > 0.5).

The ANOVA of the mean P2 amplitude at 136–176 ms over the frontal/central electrodes showed significant main effects of race [*F*(1,48) = 174.98, *P* < 0.001, η_p_^2^ = 0.79] and expression [*F*(1,48) = 33.20, *P* < 0.001, η_p_^2^ = 0.41, Table 4], indicating that the P2 amplitude was enlarged by Caucasian than Asian faces and by painful than neutral expressions. These results replicate the previous ERP findings (e.g. Ito and Bartholow, 2009; Sheng and Han, 2012) and suggest that the frontal/central P2 was involved in the coding of both racial identity and emotional states (i.e. pain). There was also a significant interaction of expression–race on the P2 amplitude [*F*(1,48) = 11.14, *P* = 0.002, η_p_^2^ = 0.19], indicating stronger effects of painful expression on the P2 amplitude in response to Asian than Caucasian faces. These results replicate the previous ERP results that indicate stronger empathic neural responses to same-race than other-race individuals (Sheng and Han, 2012; Sheng *et al.*, 2013, 2016; Li *et al.*, 2015; Han *et al.*, 2016).

**Table 4:** Mean P2 amplitudes (μV) in G/G and A/A carriers (Mean ± SE)

			G/G homozygote		A/A homozygote	
			Painful	Neutral	Painful	Neutral
P2 Amplitudes	Fellow team	Asian	4.01 ± 0.50	3.61 ± 0.43	4.82 ± 0.56	3.48 ± 0.48
		Caucasian	5.18 ± 0.53	4.67 ± 0.51	5.58 ± 0.60	5.13 ± 0.54
	Opponent team	Asian	4.41 ± 0.48	3.36 ± 0.49	4.23 ± 0.53	3.80 ± 0.54
		Caucasian	4.94 ± 0.47	4.65 ± 0.47	5.28 ± 0.52	5.14 ± 0.58

**Fig. 2:**
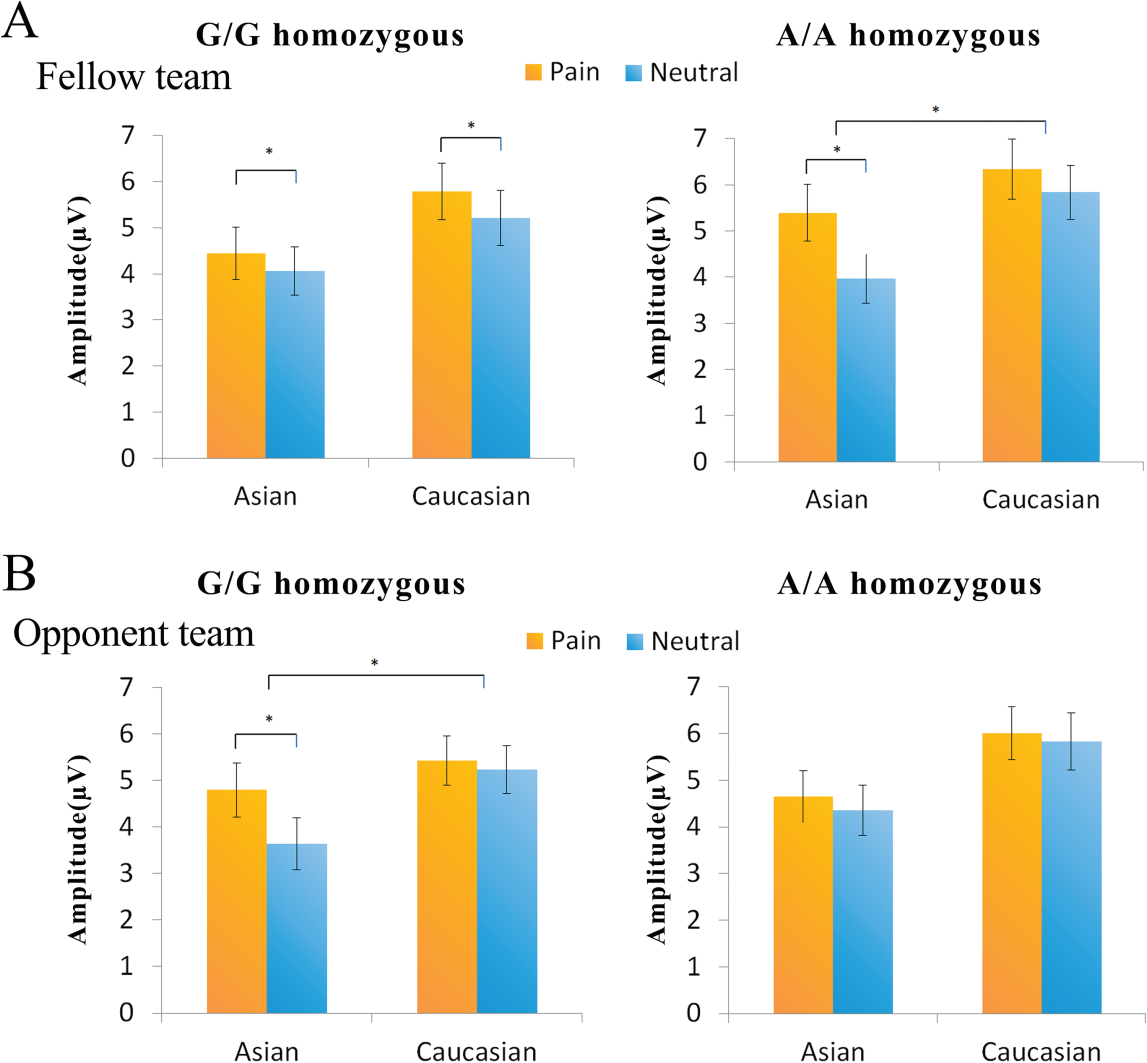
The mean P2 amplitudes at 136–176 ms in responses to faces of the fellow team (A) and to faces of the opponent team faces (B). Error bars represent the standard errors. The asterisks represent a significance level of 0.05.

Most interestingly, the ANOVA of the P2 amplitude at 136–176 ms showed a significant four-way interaction of genotype–race–expression–mini group [*F*(1,48) = 4.51, *P* = 0.039, η_p_^2^ = 0.09], indicating different patterns of modulations of empathic neural responses to others’ pain in the P2 time window by racial and mini group relationships in G/G and A/A carriers. Thus, we further analyzed the empathic neural responses (i.e. P2 amplitudes to painful *vs* neutral expressions) in the two genotype groups, respectively. For G/G carriers, ANOVAs of the P2 amplitudes to faces of the fellow team showed a significant main effect of expression [*F*(1,24) = 10.87, *P* = 0.003, η_p_^2^ = 0.31] but no significant interactions of race–expression [*F*(1,24) = 0.07, *P* = 0.790, η_p_^2^ = 0.003], indicating similar empathic neural responses in the P2 time window to Asian and Caucasian faces (Figure 2A). ANOVAs of the P2 amplitudes to faces of the opponent team, however, showed a significant interaction of race–expression [*F*(1,24) = 13.40, *P* = 0.001, η_p_^2^ = 0.36] because G/G carriers showed larger P2 amplitudes to painful than neutral expressions of Asian faces [*F*(1,24) = 20.96, *P* < 0.001, η_p_^2^ = 0.47] but not of Caucasian faces [*F*(1,24) = 1.83, *P* = 0.189, η_p_^2^ = 0.07]. These results suggest that G/G carriers showed empathic neural responses in the P2 time window to faces who shared in-group membership with the observers, as defined by either one of the two intergroup (racial or mini-group) relationships.

For A/A carriers, however, ANOVAs of the P2 amplitudes to faces of the fellow team revealed a significant interaction of race–expression [*F*(1,24) = 7.36, *P* = 0.012, η_p_^2^ = 0.24] due to that the P2 amplitude was enlarged to painful than neutral expressions of Asian faces [*F*(1,24) = 35.11, *P* < 0.001, η_p_^2^ = 0.59] but not of Caucasian faces [*F*(1,24) = 2.24, *P* = 0.148, η_p_^2^ = 0.09, Figure 2B]. ANOVAs of the P2 amplitudes to faces of the opponent team failed to show a significant interaction of race–expression [*F*(1,24) = 1.16, *P* = 0.292, η_p_^2^ = 0.05]. Moreover, the P2 amplitudes did not differ significantly between painful and neutral expressions of either Asian faces [*F*(1,24) = 3.53, *P* = 0.072, η_p_^2^ = 0.13] or Caucasian faces (F(1,24) = 0.49, p = 0.490, η_p_^2^ = 0.02). These results indicate that A/A carriers showed empathic neural responses in the P2 time window only to faces who shared in-group membership with the observers in terms of both intergroup (racial and mini group) relationships.

**Fig. 3:**
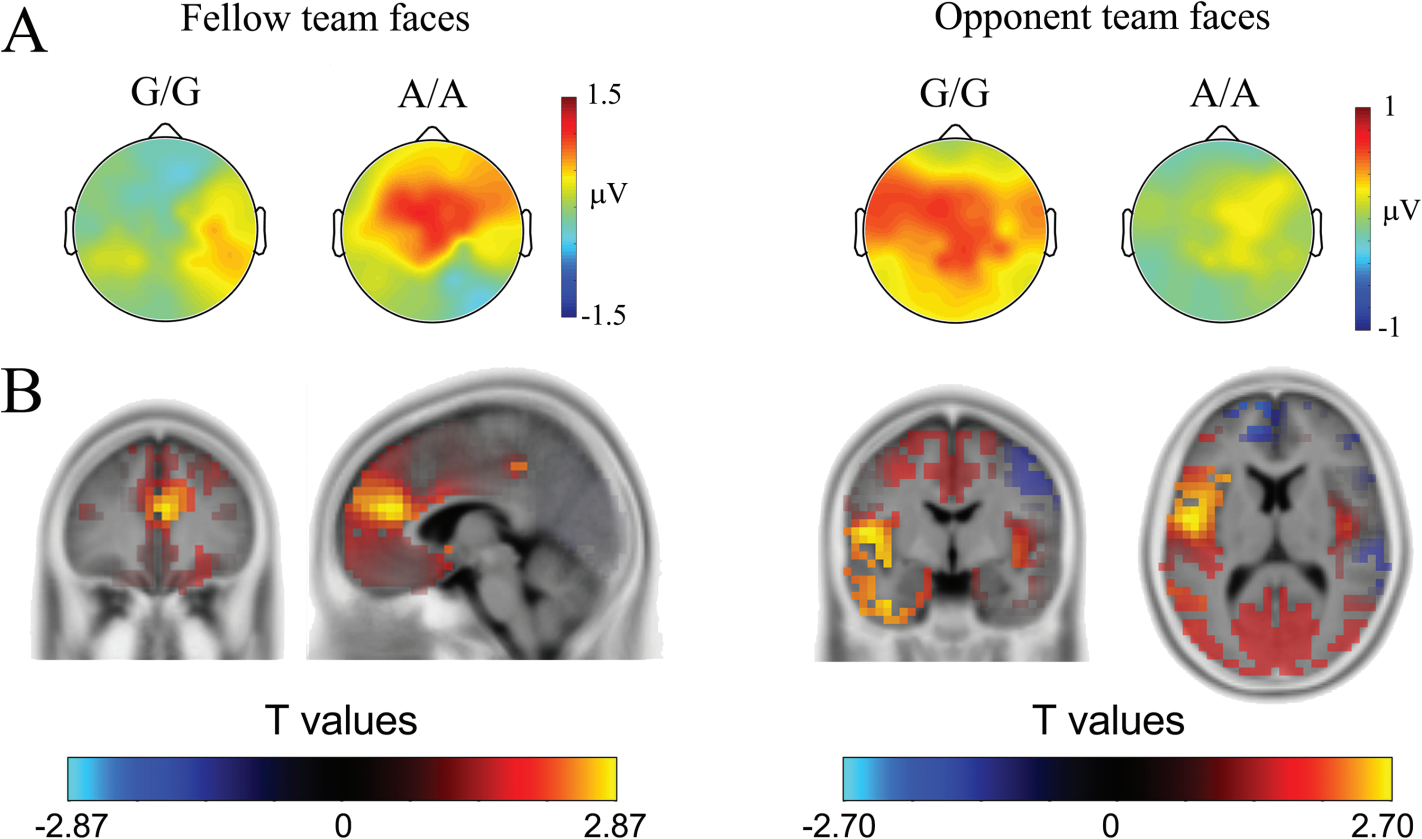
ERP results. (A) Voltage topographies show the scalp distribution of the racial in-group bias [(painful *vs* neutral expressions)_Asian faces_ minus (painful *vs* neutral expressions)_Caucasian faces_] in empathic neural responses in the P2 time window. (B) Illustration of the sources of racial in-group bias in empathic neural responses to fellow and opponent team faces in the P2 time window, with collapsed data for the genotype groups.

To examine the scalp distribution of racial in-group bias in empathic neural responses in the P2 time window, we calculated the voltage topographies of the difference wave [(painful *vs* neutral expressions)_Asian faces_ minus (painful *vs* neutral expressions)_Caucasian faces_]. As shown in Figure 3A, racial in-group bias in empathic neural responses was prominent for fellow team faces over the central/frontal region in A/A carriers but for opponent team faces over the middle and left frontal regions in G/G carriers. Consistent with the scalp distribution, source estimation using sLORETA suggested that, for faces of the fellow team, increased empathic neural responses in the P2 time window to Asian *vs* Caucasian faces had potential sources in the ACC (peak Montreal Neurological Institute (MNI) coordinates: 5, 40 and 20; Figure 3B), similar to the previous results (Sheng and Han, 2012). By contrast, for faces of the opponent team, enhanced empathic neural responses in the P2 time window to Asian *vs* Caucasian faces had potential sources in the left middle insula and inferior frontal cortex (peak MNI coordinates: −50, −5 and 10; Figure 3B).

The ANOVA of the N2 amplitudes at 200–340 ms showed significant main effects of race [*F*(1,48) = 138.21, *P* < 0.001, η_p_^2^ = 0.74] and expression [*F*(1,48) = 22.23, *P* < 0.001, η_p_^2^ = 0.32]. The N2 amplitude was larger (more negative) to Asian than Caucasian faces and was positively shifted in response to painful *vs* neutral expressions (Figure 1). These results replicate the previous ERP results (Sheng and Han, 2012; Sessa *et al.*, 2013; Sheng *et al.*, 2013). However, neither the interaction of race–expression [*F*(1,48) = 1.17, *P* = 0.284, η_p_^2^ = 0.02] nor the interaction of genotype–race–expression–mini group [*F*(1,48) = 2.06, *P* = 0.157, η_p_^2^ = 0.04] was significant, suggesting that neural responses related to race and empathy in the N2 time window were not modulated by OXTR genotype and intergroup relationship.

Given that the peak latency of the P3 component was different between G/G and A/A groups, we calculated the mean P3 amplitude at 400–600 ms for A/A group and 440–640 ms for G/G group. The ANOVA of the P3 amplitude showed neither a significant main effect of expression [*F*(1,48) = 0.19, *P* = 0.66, ƞ_p_^2^ = 0.004] nor its interaction with race [*F*(1,48) = 0.49, *P* = 0.49, ƞ_p_^2^ = 0.01]. However, there was a significant four-way interaction of genotype–race–expression–mini group [*F*(1,48) = 8.12, *P* = 0.006, ƞ_p_^2^ = 0.15]. Thus, we analyzed the P3 amplitudes in response to fellow team and opponent team faces. There was a significant interaction of genotype–race–expression for opponent team faces [*F*(1,48) = 4.70, *P* = 0.03, ƞ_p_^2^ = 0.09] but for not fellow team faces [*F*(1,48) = 2.98, *P* = 0.09, ƞ_p_^2^ = 0.06]. Further analyses of the P3 amplitudes to opponent team faces revealed that G/G carriers showed larger P3 amplitudes to painful than neutral expressions of Asian faces [*F*(1,24) = 7.47, *P* = 0.01, ƞ_p_^2^ = 0.24] but of not Caucasian faces [*F*(1,24) = 0.28, *P* = 0.60, ƞ_p_^2^ = 0.01]. Neither the main effect of expression nor the interaction of race–expression was significant for A/A carriers [*F*(1,24) = 2.85 and 0.72, *P* = 0.10 and 0.40, ƞ_p_^2^ = 0.11 and 0.03].These results suggest that G/G carriers but not A/A carriers showed empathic neural responses in the P3 time window to opponent team faces with whom the observers shared racial identity.

### Empathy trait, ethnic identity and racial in-group bias in empathic neural responses

**Fig. 4:**
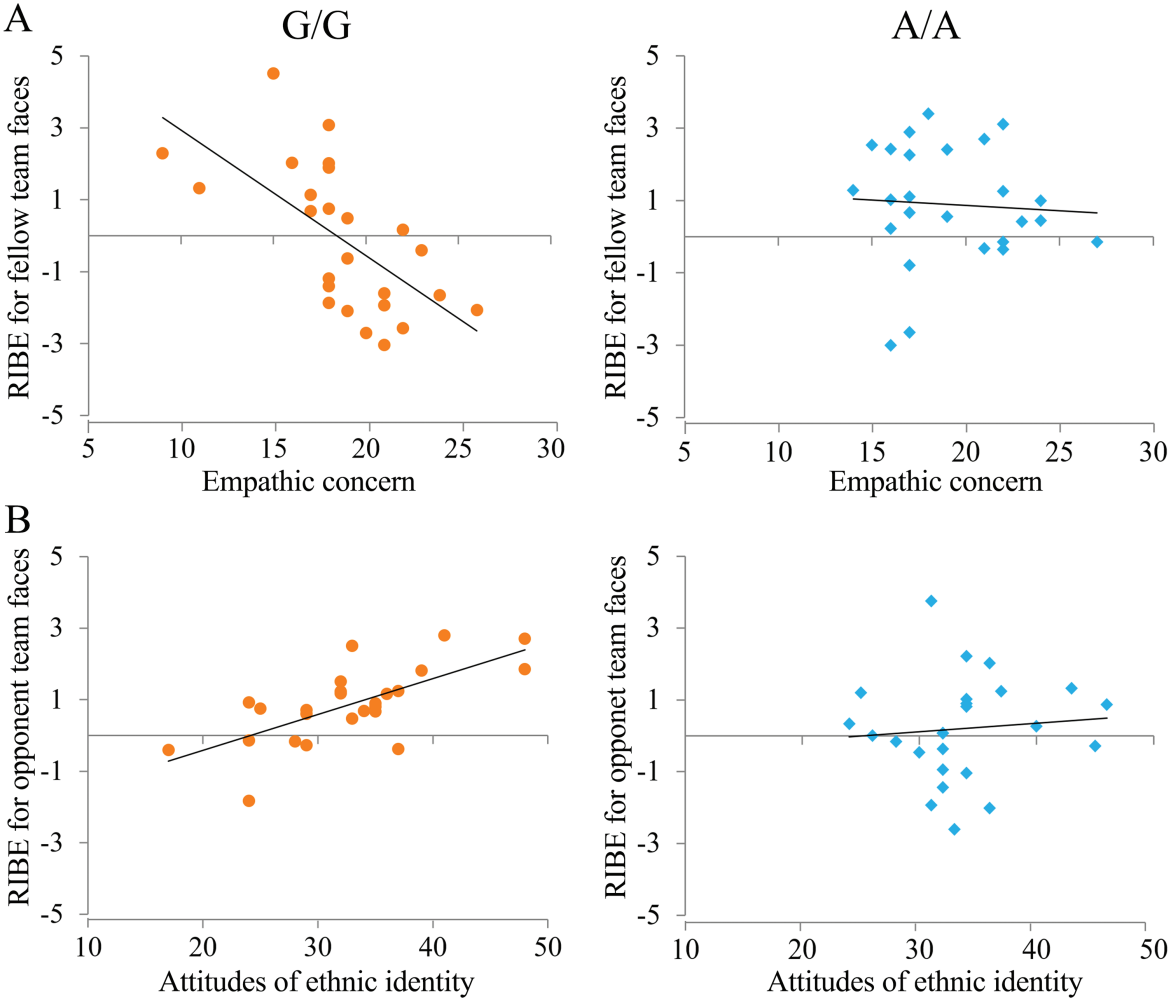
Variations of RIBE indexed by empathic neural responses in the P2 time window [(painful *vs* neutral expressions)_Asian faces_ minus (painful *vs* neutral expressions)_Caucasian faces_] across individuals’ empathic concern and ethnic identity. (A) RIBE in response to fellow team faces in the P2 amplitudes negatively correlated with empathic concern scores for G/G but not A/A carriers. (B) RIBE indexed by empathic neural response to opponent team faces in the P2 amplitude positively correlated with rating scores of ethnic identity for G/G but not A/A carriers.

Next we tested whether racial in-group bias in empathic neural responses in the P2 time window varies across individuals’ empathy traits. Behavioral studies have suggested that the empathic concern and perspective taking subscales of IRI correspond more directly to the conceptual definitions of empathy and the empathic concern subscale tends to reflect the affective component of empathy whereas the perspective taking subscale tends to reflect the cognitive component of empathy (Bohart *et al.*, 2002; Ridley and Lingle, 1996). Moreover, our previous studies revealed that the P2 amplitude to pain (*vs* neutral) expression was positively associated with the empathic concern score (Sheng and Han, 2012; Sheng *et al.*, 2013). Thus, we calculated correlations between empathic concern scores and racial in-group bias in differential P2 amplitudes to painful *vs* neutral expressions of fellow and opponent team faces, respectively. These analyses revealed significant correlations between empathic concern scores and racial in-group bias in empathic neural responses in the P2 time window to fellow team faces [*r*(50) = −0.36, *P* = 0.011] but not to opponent team faces [*r*(50) = −0.07, *P* = 0.629]. The results suggested that participants with higher empathic concern scores showed decreased racial in-group bias in empathic neural responses to fellow team faces in the P2 time window. Interestingly, separate analyses showed that the association between empathic concern scores and racial in-group bias in empathic neural responses to fellow team faces in the P2 time window was significant for G/G carriers [*r*(25) = −0.62, *P* = 0.001] but not for A/A carriers [*r*(25) = −0.09, *P* = 0.663, Figure 4A]. This difference in association patterns between G/G and A/A carriers was further confirmed by transforming r values to *z* scores [Fisher *r* to *z*: *z* = 2.11, *P* = 0.035). Separate analyses further revealed that higher empathic concerns scores predicted greater empathic neural responses in the P2 time window to Caucasian faces of the fellow team for G/G carriers [*r*(25) = 0.46, *P* = 0.022], but not for A/A carriers [*r*(25) = −0.14, *P* = 0.505]. The association patterns were also significantly different between G/G and A/A carriers (Fisher *r* to *z*: *z* = 2.12, *P* = 0.034).

Finally, we examined whether racial in-group bias in empathic neural responses varies across individuals’ self-reported ethnic identity. We calculated the correlations between ethnic identity scores and racial in-group bias in empathic neural responses in the P2 time window for fellow team and opponent team faces, respectively. These analyses revealed significant correlations between ethnic identity scores and racial in-group bias in neural responses in the P2 time window to opponent team faces [*r*(50) = 0.31, *P* = 0.029] but not to fellow team faces [*r*(50) = −0.07, *P* = 0.642]. Stronger ethnic identity predicted greater racial in-group bias in neural responses to opponent team faces. Separate analysis further showed that the association between ethnic identity scores and racial in-group bias in neural responses in the P2 time window was significant for G/G carriers [*r*(25) = 0.62, *P* = 0.001] but not for A/A carriers [*r*(25) = 0.10, p = 0.638, Figure 4B). The difference in association patterns between G/G and A/A carriers was further confirmed by transforming r values to z scores (Fisher r to z: z = 2.07, p = 0.039). Separate analysis further showed that stronger ethnic identity predicted greater empathic responses to Asian faces of the opponent team in the P2 time window for G/G carriers (r(25) = 0.54, *P* = 0.005] but not for A/A carriers [*r*(25) = −0.04, *P* = 0.834]. The association patterns were significantly different between G/G and A/A carriers [Fisher *r* to *z*: *z* = 2.14, *P* = 0.032].

## Discussion

The present study investigated whether and how OXTR interacts with intergroup relationships to modulate empathic brain activity by recording ERPs to painful and neutral expressions of same-race and other-race faces from G/G and A/A carriers of OXTR. The ERP results focused on the P2 amplitude because previous work has shown that the P2 amplitude was enhanced by painful *vs* neutral expressions and the enhancement of the P2 amplitude predicted subjective feelings of others’ pain (Sheng and Han, 2012; Sheng *et al.*, 2013, 2016; Li *et al.*, 2015; Han *et al.*, 2016). Similar to the previous research (e.g. Sheng and Han, 2012), we found a greater P2 amplitude to painful *vs* neutral expressions and this empathic neural response was stronger to same-race than other-race faces across the whole sample, providing further evidence for RIBE in early neural responses to others’ pain. Source estimation suggested that the increased P2 responses to same-race than other-race pain might arise from the ACC and anterior insula that have been demonstrated to be involved in empathy for pain in previous fMRI studies (Singer *et al.*, 2004; Jackson *et al.*, 2005; Han *et al.*, 2009; Fan *et al.*, 2011; Lamm *et al.*, 2011; Luo *et al.*, 2015a). Most importantly, as predicted, we found that empathic neural responses in the P2 time window showed different patterns of modulations by racial and mini group relationships in G/G and A/A carriers of OXTR. Our ERP results can be understood within a framework that assumes greater flexibility of empathy for pain in the context of in-group memberships in G/G (*vs* A/A) carriers of OXTR.

Specifically, using the minimal group manipulations (Van Bavel *et al.*, 2008; Sheng and Han, 2012) that produced fellow and opponent teams consisting of both same-race and other-race faces, we found that G/G carriers of OXTR exhibited empathic neural responses in the P2 time window to same-race faces, regardless of whether these faces were from the fellow or opponent team. G/G carriers also showed significant empathic neural responses in the P2 time window to other-race faces from the fellow team. These individuals did not show empathic neural responses in the P2 time window only to other-race faces from the opponent team. These results indicate that, for G/G carriers, mini group memberships can override the interracial relationships that lead to decreased empathic neural responses to other-race individuals as shown in previous research (Xu *et al.*, 2009; Avenanti *et al.*, 2010; Mathur *et al.*, 2010; Cheon *et al.*, 2011; Sheng and Han, 2012; Azevedo *et al.*, 2013; Contreras-Huerta *et al.*, 2013; Sheng *et al.*, 2013, 2016; Sessa *et al.*, 2014; Li *et al.*, 2015; Han *et al.*, 2016, Fourie *et al.*, 2017; also see Han, 2015, 2018). Consequently, the racial in-group bias in empathic neural responses in the P2 time window was reduced by in-group membership built through mini group manipulations. These results indicate that G/G carriers of OXTR showed empathic neural responses, as indexed by increased P2 amplitude to painful *vs* neutral expressions, to those whom were perceived as in-group members, as defined by either racial or mini group relationships.

A/A carriers of OXTR, however, showed different patterns of modulation of empathic neural responses in the P2 time window by interracial and mini group relationships. A/A carriers did not show empathic neural responses in the P2 time window to other-race faces from either the fellow or the opponent team. Furthermore, A/A carriers even failed to show P2 empathic neural responses to same-race faces of the opponent team. It seemed that only one (racial or mini group) in-group membership was not enough to generate significant empathic responses to others’ pain in A/A carriers. A/A carriers showed P2 empathic neural responses only to those with whom they shared both racial and mini group memberships (i.e. same-race faces from the fellow team). As a result, the P2 amplitude recorded from A/A carriers showed a racial in-group bias in responses to perceived pain of faces from the fellow but not opponent team. These patterns are obviously different from those observed in G/G carriers.

The results of P2 empathic neural responses support our hypothesis that G/G carriers of OXTR are sensitive to in-group memberships, as defined by either racial or mini group relationships, and thus they showed significant empathic neural responses to perceived pain of in-group members defined by either or both of the two in-group relationships (i.e. individuals of same-race or fellow team). By contrast, A/A carrier are less flexible to social relationships and they showed empathic neural responses only to those with whom they shared in-group membership based on both racial and mini group relationships. Multiple psychological processes might contribute to the distinct patterns of the interaction of racial and mini group memberships on the P2 amplitude to painful *vs* neutral expressions between G or A allele carriers of OXTR rs53576. For example, as oxytocin is implicated in the ability to recognize emotions (Domes *et al.*, 2007; Di Simplicio *et al.*, 2009; Rodrigues *et al.*, 2009; Marsh *et al.*, 2010) and G compared to A allele carriers of OXTR are more sensitive to others’ emotional states (Bakermans-Kranenburg and van IJzendoorn, 2008), the P2 results observed here might arise from the difference in emotion recognition between the two genetic types since painful expressions were used in our work. There are also findings suggesting associations between oxytocin and trust/motivation to engage socially (Kosfeld *et al.*, 2005; Baumgartner *et al.*, 2008) and G compared to A carriers showed higher trust behavior (Krueger *et al.*, 2012). Thus, the difference in social trust and motivation between G and A carriers of OXTR may also influence the degree to which group identity (e.g. in-group or out-group members) is constructed that further modulates the P2 responses to perceived pain in others. These analyses fit with a broader role of oxytocin in social cognition and behavior and can be tested in future work.

The results of our source estimation suggested that the racial in-group bias in empathic neural responses in the P2 time window might originate from the ACC for faces of the fellow team but from the left middle insula and inferior frontal cortex for faces of the opponent team. Although these results did not differ significantly between G/G and A/A carriers, our previous fMRI research found that empathic neural responses in G/G carriers of OXTR were characterized by a stronger racial in-group bias in ACC activity in response to perceived pain compared to that observed in A/A carriers (Luo *et al.*, 2015a). The ACC and insula are the core regions of the empathy network, and the ACC is recruited mainly in the cognitive component of empathy whereas the insula is activated in both cognitive–evaluative and affective–perceptual forms of empathy (Fan *et al.*, 2011). In line with these perspectives, one may speculate that the racial bias in empathy for the opponent team members’ pain may occur in the affective domains whereas viewing painful expressions of other-race individuals of the fellow team is mainly associated with strong cognitive conflict. However, how these distinct patterns of empathy modulations are associated with OXTR remains unclear and should clarified in future work.

The distinct patterns of P2 empathic neural responses observed in G/G and A/A carriers cannot be attributed to differences in age, empathy ability or attitude toward same-race/other-race faces because these were matched in the two genotype groups in our study. However, we found that RIBE indexed by the empathic neural responses in the P2 time window varied significantly across individuals’ empathic concern and ethnic identity for G/G but not for A/A carriers. Specifically, G/G carriers who reported greater empathic concern showed less RIBE in the P2 response to fellow team faces. Moreover, G/G carriers with stronger ethnic identity showed larger RIBE in P2 response to opponent team faces. These results have two implications. First, RIBE is more sensitive to individuals’ empathic traits and ethnic identity in G/G compared to A/A carriers of OXTR rs53576. This finding implicates that the modulation of empathic neural responses by racial relationships is not fully determined by an individual’s genetic makeup. Individuals’ empathy traits and self-recognized ethnic identity also influence RIBE, although these effects seem to be more salient in G/G carriers. Second, the correlation results of G/G carriers suggest that individuals’ with higher empathy ability empathized with fellow team members’ pain regardless of their ethnic identities and thus showed reduced RIBE. In addition, individual G/G carriers with greater ethnic identity may discriminate same-race and other-race individuals of the opponent team more strongly and thus empathized with same-race pain more strongly to produce RIBE. These results provide electrophysiological evidence for variations in RIBE along empathic traits and ethnic identity and raise an important question for future research, i.e. why do G/G but not A/A carriers show RIBE variations along empathic traits and ethnic identity?

The distinct patterns of empathic neural responses observed in G/G and A/A carriers were observed for the early P2 component but not for the following N2 component. The P3 amplitude in the current work failed to show a significant main effect of facial expression although only G/G carriers showed larger P3 amplitudes in response to same-race faces from the opponent team. Previous studies have shown that both the N2 and P3 amplitude were modulated by painful *vs* neutral expressions (also see Sheng and Han, 2012; Sheng *et al.*, 2013; Sessa *et al.*, 2013), but these effects were more salient when participants focused their attention on emotional states of target faces compared to target faces’ racial identity (Sheng and Han, 2012). It has been suggested that the early neural responses to others’ pain are involved in coding and sharing emotional states of others and underlay subjective feeling of both others’ pain and self-unpleasantness whereas the late neural responses to others’ pain reflect enhanced evaluation and appraisal process during empathy (Fan and Han, 2008). Our ERP results implicate that interactions between OXTR and intergroup relationship may occur during early emotional encoding and sharing when perceiving others’ suffering, although the results of the P3 amplitude also suggest that G/G carriers might engage slightly enhanced cognitive evaluation of painful same-race faces from the opponent team. Because the race judgment task emphasizes racial identity but not emotional states of each individual face (Sheng and Han, 2002), our work allowed estimation of automatic empathic responses rather than top–down cognitive component of empathy and thus did not allow us to explore the entire temporal courses of the empathic neural responses in G/G and A/A carriers. Future research may test this using the pain judgment task (Sheng and Han, 2012). Future research should also examine whether the modulation of neural responses in the P2 time window contributes to previous findings that GG carriers of OXTR rs53576 were more sensitive to other socio-emotional signals than A/A carriers (Lucht *et al.*, 2009; Rodrigues *et al.*, 2009; Smith *et al.*, 2014).

A recent ERP study found that male G/G relative to A/A carriers of OXTR rs53576 showed larger N1 amplitudes in response to images of both humans and objects and larger N2 amplitudes in response to images of humans but not objects (Choi *et al.*, 2017). These findings have been proposed to indicate that OXTR rs53576 may affect emotional processing of both social and nonsocial cues. The current work tested female G/G and A/A carriers of OXTR rs53576 and did not find genotype differences in N2 amplitudes in response to faces. It is thus likely that the influences of OXTR rs53576 on emotional processing may differ between males and females. Future research should further investigate whether similar patterns of interactions between OXTR and intergroup relationship on empathic neural responses can be observed in male participants given gender differences in brain activity in response to nasal administration of oxytocin (e.g. Ma *et al.*, 2016).

Finally, a previous fMRI study found greater activity in the amygdala, fusiform gyri, orbitofrontal cortex and dorsal striatum when participants viewed novel in-group faces than when they viewed novel out-group faces even though both the novel in-group and the novel out-group consisted of half same-race and half other-race faces (Van Bavel *et al.*, 2008). These findings suggest that mini group identity can override racial identity in the modulation of brain activities related to perceptual and emotional processing of faces. Given our ERP findings of interactions between OXTR and intergroup relationship on empathic neural responses, future research should take participants’ genetic makeup into consideration when examining the role of group identity in modulations of brain activity underlying social cognition and behavior.

In conclusion, while our previous studies discovered that both sociocultural experiences (Zuo and Han, 2013) and physical environments (Luo *et al.*, 2018) can influence RIBE, the current work further revealed ERP evidence for interactions between OXTR and intergroup relationships on empathy for same-race and other-race individuals’ pain. Our ERP results suggest that empathic neural responses are more sensitive to social relationships between observers and targets and to individuals’ empathy trait and ethnic identity in G/G than A/A carriers of OXTR rs53576. Modulations of empathic neural responses to others’ suffering and relevant pro-social behavior by both sociocultural experience and genetic makeup may help individuals adapt to social environments.

## Funding

This work was supported by the National Natural Science Foundation of China (31661143039, 31421003 and 31800916), Natural Science Foundation of Guangdong Province (Project 2017A030310553), Science Foundation of Ministry of Education of China (Project 17YJCZH121) and German Research Foundation (Project HE 4566/2-1).

## References

[ref1] Avenanti A., Sirigu A., Aglioti S.M. (2010). Racial bias reduces empathic sensorimotor resonance with other-race pain. Current Biology, 20(11), 1018–22. DOI: 10.1016/j.cub.2010.03.071. 20537539

[ref2] Azevedo R.T., Macaluso E., Avenanti A., Santangelo V., Cazzato V., Aglioti S.M. (2013). Their pain is not our pain: brain and autonomic correlates of empathic resonance with the pain of same and different race individuals. Human Brain Mapping, 34(12), 3168–81. DOI: 10.1002/hbm.22133. 22807311 PMC6870096

[ref3] Bakermans-Kranenburg M.J., van IJzendoorn M.H. (2008). Oxytocin receptor (OXTR) and serotonin transporter (5-HTT) genes associated with observed parenting. Social Cognitive and Affective Neuroscience, 3(2), 128–34. DOI: 10.1093/scan/nsn004. 19015103 PMC2555463

[ref4] Baumgartner T., Heinrichs M., Vonlanthen A., Fischbacher U., Fehr E. (2008). Oxytocin shapes the neural circuitry of trust and trust adaptation in humans. Neuron, 58(4), 639–50 10.1016/j.neuron.2008.04.009. 18498743

[ref5] Bohart A.C., Elliot R., Greenberg L.S., Watson J.C. (2002). Empathy In: Norcross J.C., editor. Psychotherapy Relationships that Work: Therapist Contributions and Responsiveness to Patients, London: Oxford University Press, 89–108.

[ref6] Brown D.E. (1991). Human Universals, New York: McGraw-Hill.

[ref7] Cheon B.K., Im D.M., Harada T., Kim J.S., et al. (2011). Cultural influences on neural basis of intergroup empathy. NeuroImage, 57(2), 642–50. DOI: 10.1016/j.neuroimage.2011.04.031. 21549201

[ref8] Choi D., Minote N., Watanuki S. (2017). Associations between the oxytocin receptor gene (OXTR) rs53576 polymorphism and emotional processing of social and nonsocial cues: an event-related potential (ERP) study. Journal of Physiological Anthropology, 36(1), 12 DOI: 10.1186/s40101-016-0125-3. 28126018 PMC5270231

[ref9] Cikara M., Van Bavel J.J. (2014). The neuroscience of intergroup relations: an integrative review. Perspectives on Psychological Science, 9(3), 245–74. DOI: 10.1177/1745691614527464. 26173262

[ref10] Cikara M., Bruneau E.G., Saxe R.R. (2011a). Us and them: intergroup failures of empathy. Current Directions in Psychological Science, 20(3), 149–53. DOI: 10.1016/j.jesp.2014.06.007.

[ref11] Cikara M., Botvinick M.M., Fiske S.T. (2011b). Us versus them: social identity shapes neural responses to intergroup competition and harm. Psychological Science, 22(3), 306–13. DOI: 10.1177/0956797610397667. 21270447 PMC3833634

[ref12] Contreras-Huerta L.S., Baker K.S., Reynolds K.J., Batalha L., Cunnington R. (2013). Racial bias in neural empathic responses to pain. PLoS One, 8(12), e84001 DOI: 10.1371/journal.pone.0084001. 24376780 PMC3871655

[ref13] Contreras-Huerta L.S., Hielscher E., Sherwell C.S., Rens N., Cunnington R. (2014). Intergroup relationships do not reduce racial bias in empathic neural responses to pain. Neuropsychologia, 64, 263–70. DOI: 10.1016/j.neuropsychologia.2014.09.045. 25281885

[ref14] Davis M.H. (1983). Measuring individual differences in empathy: evidence for a multidimensional approach. Journal of Personality and Social Psychology, 44(1), 113–26. DOI: 10.1037//0022-3514.44.1.113.

[ref15] De Dreu C.K., Greer L.L., Handgraaf M.J., Shalvi S., et al. (2010). The neuropeptide oxytocin regulates parochial altruism in intergroup conflict among humans. Science, 328(5984), 1408–11. DOI: 10.1126/science.1189047. 20538951

[ref16] Decety J., Jackson P.L. (2004). The functional architecture of human empathy. Behavioral and Cognitive Neuroscience Reviews, 3(2), 71–100. DOI: 10.1177/1534582304267187. 15537986

[ref17] Decety J., Bartal I.B.A., Uzefovsky F., Knafo-Noam A. (2016). Empathy as a driver of prosocial behaviour: highly conserved neurobehavioural mechanisms across species. Philosophical Transactions of the Royal Society B, 371(1686), 20150077 DOI: 10.1098/rstb.2015.0077. PMC4685523 26644596

[ref18] Di Simplicio M., Massey-Chase R., Cowen P.J., Harmer C.J. (2009). Oxytocin enhances processing of positive versus negative emotional information in healthy male volunteers. Journal of Psychopharmacology, 23(3), 241–8. DOI: 10.1177/0269881108095705. 18801829

[ref19] Domes G., Heinrichs M., Gläscher J., Büchel C., Braus D.F., Herpertz S.C. (2007). Oxytocin attenuates amygdala responses to emotional faces regardless of valence. Biological Psychiatry, 62(10), 1187–90. DOI: 10.1016/j.biopsych.2007.03.025. 17617382

[ref20] Eres R., Molenberghs P. (2013). The influence of group membership on the neural correlates involved in empathy. Frontiers in Human Neuroscience, 7, 176 DOI: 10.3389/fnhum.2013.00176. 23653604 PMC3644680

[ref21] Fan Y., Han S. (2008). Temporal dynamic of neural mechanisms involved in empathy for pain: an event-related brain potential study. Neuropsychologia, 46(1), 160–73. DOI: 10.1016/j.neuropsychologia.2007.07.023. 17825852

[ref22] Fan Y., Duncan N.W., de Greck M., Northoff G. (2011). Is there a core neural network in empathy? An fMRI based quantitative meta-analysis. Neuroscience and Biobehavioral Reviews, 35(3), 903–11. DOI: 10.1016/j.neubiorev.2010.10.009. 20974173

[ref23] Faul F., Erdfelder E., Lang A.G., Buchner A. (2007). G* power 3: a flexible statistical power analysis program for the social, behavioral, and biomedical sciences. Behavior Research Methods, 39(2), 175–91. DOI: 10.3758/BF03193146. 17695343

[ref24] Fourie M.M., Stein D.J., Solms M., Gobodo-Madikizela P., Decety J. (2017). Empathy and moral emotions in post-apartheid South Africa: an fMRI investigation. Social Cognitive and Affective Neuroscience, 12(6), 881–92. DOI: 10.1093/scan/nsx019. 28338783 PMC5472164

[ref25] Greenwald A.G., McGhee D.E., Schwartz J.L. (1998). Measuring individual differences in implicit cognition: the implicit association test. Journal of Personality and Social Psychology, 74(6), 1464 DOI: 10.1037//0022-3514.74.6.1464. 9654756

[ref26] Greenwald A.G., Nosek B.A., Banaji M.R. (2003). Understanding and using the implicit association test: I. an improved scoring algorithm. Journal of Personality and Social Psychology, 85(2), 197 DOI: 10.1037/h0087889. 12916565

[ref27] Han S. (2015). Intergroup relationship and empathy for others’ pain: a social neuroscience approach Neuroscience in Intercultural Contexts, New York, NY: Springer, 31–47. DOI: 10.1007/978-1-4939-2260-4_2.

[ref28] Han S. (2018). Neurocognitive basis of racial ingroup bias in empathy. Trends in Cognitive Sciences, 22(5), 400–21. DOI: 10.1016/j.tics.2018.02.013. 29563059

[ref29] Han S., Fan Y., Xu X., et al. (2009). Empathic neural responses to others’ pain are modulated by emotional contexts. Human Brain Mapping, 30(10), 3227–37. DOI: 10.1002/hbm.20742. 19235883 PMC6870998

[ref30] Han X., Luo S., Han S. (2016). Embodied neural responses to others’ suffering. Cognitive Neuroscience, 7(1–4), 114–27. DOI: 10.1080/17588928.2015.1053440. 26111085

[ref31] Hein G., Silani G., Preuschoff K., Batson C.D., Singer T. (2010). Neural responses to ingroup and outgroup members' suffering predict individual differences in costly helping. Neuron, 68(1), 149–60. DOI: 10.1016/j.neuron.2010.09.003. 20920798

[ref32] Ito T.A., Bartholow B.D. (2009). The neural correlates of race. Trends in Cognitive Sciences, 13(12), 524–31. DOI: 10.1016/j.tics.2009.10.002. 19896410 PMC2796452

[ref33] Jackson P.L., Meltzoff A.N., Decety J. (2005). How do we perceive the pain of others? A window into the neural processes involved in empathy. NeuroImage, 24(3), 771–9. DOI: 10.1016/j.neuroimage.2004.09.006. 15652312

[ref34] Kogan A., Saslow L.R., Impett E.A., Oveis C., Keltner D., Saturn S.R. (2011). Thin-slicing study of the oxytocin receptor (OXTR) gene and the evaluation and expression of the prosocial disposition. Proceedings of the National Academy of Sciences, 108(48), 19189–92. DOI: 10.1073/pnas.1112658108. PMC3228468 22084107

[ref35] Kosfeld M., Heinrichs M., Zak P.J., Fischbacher U., Fehr E. (2005). Oxytocin increases trust in humans. Nature, 435(7042), 673–6. 15931222 10.1038/nature03701

[ref36] Krueger F., Parasuraman R., Iyengar V., et al. (2012). Oxytocin receptor genetic variation promotes human trust behavior. Frontiers in Human Neuroscience, 6, 4 DOI: 10.3389/fnhum.2012.00004. 22347177 PMC3270329

[ref37] Kurzban R., Neuberg S. (2005). Managing ingroup and outgroup relationships In: The Handbook of Evolutionary Psychology, New York: John Wiley & Sons, Inc.

[ref38] Lamm C., Decety J., Singer T. (2011). Meta-analytic evidence for common and distinct neural networks associated with directly experienced pain and empathy for pain. NeuroImage, 54(3), 2492–502. DOI: 10.1016/j.neuroimage.2010.10.014. 20946964

[ref39] Li X., Liu Y., Luo S., Wu B., Wu X., Han S. (2015). Mortality salience enhances racial in-group bias in empathic neural responses to others’ suffering. NeuroImage, 118, 376–85. DOI: 10.1016/j.neuroimage.2015.06.023. 26074201

[ref40] Lucht M.J., Barnow S., Sonnenfeld C., Rosenberger A., et al. (2009). Associations between the oxytocin receptor gene (OXTR) and affect, loneliness and intelligence in normal subjects. Progress in Neuro-Psychopharmacology and Biological Psychiatry, 33(5), 860–6. DOI: 10.1016/j.pnpbp.2009.04.004. 19376182

[ref41] Luck S.J., Gaspelin N. (2017). How to get statistically significant effects in any ERP experiment (and why you shouldn't). Psychophysiology, 54, 146–57 10.1111/psyp.12639. 28000253 PMC5178877

[ref42] Luo S., Li B., Ma Y., Zhang W., Rao Y., Han S. (2015a). Oxytocin receptor gene and racial ingroup bias in empathy-related brain activity. NeuroImage, 110, 22–31 10.1016/j.neuroimage.2015.01.042. 25637390

[ref43] Luo S., Ma Y., Liu Y., et al. (2015b). Interaction between oxytocin receptor polymorphism and interdependent culture on human empathy. Social Cognitive and Affective Neuroscience, 10, 1273–81 10.1093/scan/nsv019. 25680993 PMC4560951

[ref44] Luo S., Han X., Du N., Han S. (2018). Physical coldness enhances racial in-group bias in empathy: electrophysiological evidence. Neuropsychologia, 116, 117–25 10.1016/j.neuropsychologia.2017.05.002. 28478242

[ref45] Ma Y., Shamay-Tsoory S., Han S., Zink C.F. (2016). Oxytocin and social adaptation: insights from neuroimaging studies of healthy and clinical populations. Trends in Cognitive Sciences, 20(2), 133–45 10.1016/j.tics.2015.10.009. 26616296

[ref46] Marsh A.A., Henry H.Y., Pine D.S., Blair R.J.R. (2010). Oxytocin improves specific recognition of positive facial expressions. Psychopharmacology, 209(3), 225–32. 20186397 10.1007/s00213-010-1780-4

[ref47] Mathur V.A., Harada T., Lipke T., Chiao J.Y. (2010). Neural basis of extraordinary empathy and altruistic motivation. NeuroImage, 51(4), 1468–75 10.1016/j.neuroimage.2010.03.025. 20302945

[ref48] Molenberghs P. (2013). The neuroscience of in-group bias. Neuroscience and Biobehavioral Reviews, 37(8), 1530–6 10.1016/j.neubiorev.2013.06.002. 23769813

[ref49] Pascual-Marqui R.D. (2002). Standardized low-resolution brain electromagnetic tomography (sLORETA): technical details. Methods and Findings in Experimental and Clinical Pharmacology, 24, Suppl D, 5–12. 12575463

[ref50] Phinney J.S. (1992). The multigroup ethnic identity measure: a new scale for use with diverse groups. Journal of Adolescent Research, 7(2), 156–76 10.1177/074355489272003.

[ref51] Ridley C.R., Lingle D.W. (1996). Cultural empathy in multicultural counseling: a multidimensional process model In: Pedersen P.B., Draguns J.G., Lonner W.J., Trimble J.E., editors. Counseling Across Cultures, 4th edn, Thousand Oaks, CA: Sage, 21–46.

[ref52] Rodrigues S.M., Saslow L.R., Garcia N., John O.P., Keltner D. (2009). Oxytocin receptor genetic variation relates to empathy and stress reactivity in humans. Proceedings of the National Academy of Sciences, 106(50), 21437–41 10.1073/pnas.0909579106. PMC2795557 19934046

[ref53] Sessa P., Meconi F., Castelli L., Dell’Acqua R. (2013). Taking one’s time in feeling other-race pain: an event-related potential investigation on the time-course of cross-racial empathy. Social Cognitive and Affective Neuroscience, 9(4), 454–63 10.1093/scan/nst003. 23314008 PMC3989124

[ref54] Sessa P., Meconi F., Han S. (2014). Double dissociation of neural responses supporting perceptual and cognitive components of social cognition: evidence from processing of others' pain. Scientific Reports, 4, 7424 10.1038/srep07424. PMC4262888 25502570

[ref55] Shen F., Hu Y., Fan M., Wang H., Wang Z. (2018). Racial bias in neural response for pain is modulated by minimal group. Frontiers in Human Neuroscience, 11, 661 10.3389/fnhum.2017.00661. 29379429 PMC5770956

[ref56] Sheng F., Han S. (2012). Manipulations of cognitive strategies and intergroup relationships reduce the racial bias in empathic neural responses. NeuroImage, 61(4), 786–97 10.1016/j.neuroimage.2012.04.028. 22542636

[ref57] Sheng F., Liu Y., Zhou B., Zhou W., Han S. (2013). Oxytocin modulates the racial bias in neural responses to others’ suffering. Biological Psychology, 92(2), 380–6 10.1016/j.biopsycho.2012.11.018. 23246533

[ref58] Sheng F., Liu Q., Li H., Fang F., Han S. (2014). Task modulations of racial bias in neural responses to others' suffering. NeuroImage, 88, 263–70 10.1016/j.neuroimage.2013.10.017. 24135167

[ref59] Sheng F., Han X., Han S. (2016). Dissociated neural representations of pain expressions of different races. Cerebral Cortex, 26(3), 1221–33 10.1093/cercor/bhu314. 25576533

[ref60] Singer T., Seymour B., O'doherty J., Kaube H., Dolan R.J., Frith C.D. (2004). Empathy for pain involves the affective but not sensory components of pain. Science, 303(5661), 1157–62 DOI: 10.1126/science.1093535. 14976305 10.1126/science.1093535

[ref61] Smith K.E., Porges E.C., Norman G.J., Connelly J.J., Decety J. (2014). Oxytocin receptor gene variation predicts empathic concern and autonomic arousal while perceiving harm to others. Social Neuroscience, 9(1), 1–9 10.1080/17470919.2013.863223. 24295535 PMC3923324

[ref62] Tajfel H. (1982). Social psychology of intergroup relations. Annual Review of Psychology, 33(1), 1–39 10.1146/annurev.ps.33.020182.000245.

[ref63] Tost H., Kolachana B., Hakimi S., et al. (2010). A common allele in the oxytocin receptor gene (OXTR) impacts prosocial temperament and human hypothalamic-limbic structure and function. Proceedings of the National Academy of Sciences, 107(31), 13936–41 10.1073/pnas.1003296107. PMC2922278 20647384

[ref64] Van Bavel J.J., Packer D.J., Cunningham W.A. (2008). The neural substrates of in-group bias: a functional magnetic resonance imaging investigation. Psychological Science, 19(11), 1131–9 10.1111/j.1467-9280.2008.02214.x. 19076485

[ref65] Van IJzendoorn M.H., Bakermans-Kranenburg M.J. (2012). A sniff of trust: meta-analysis of the effects of intranasal oxytocin administration on face recognition, trust to in-group, and trust to out-group. Psychoneuroendocrinology, 37(3), 438–43 10.1016/j.psyneuen.2011.07.008. 21802859

[ref66] Xu X., Zuo X., Wang X., Han S. (2009). Do you feel my pain? Racial group membership modulates empathic neural responses. Journal of Neuroscience, 29(26), 8525–9 10.1523/JNEUROSCI.2418-09.2009. 19571143 PMC6665679

[ref67] Zuo X., Han S. (2013). Cultural experiences reduce racial bias in neural responses to others’ suffering. Culture and Brain, 1(1), 34–46 10.1007/s40167-013-0002-4.

